# Diagnostic potential of Type VII Collagen during oral carcinogenesis

**DOI:** 10.1590/1678-7757-2022-0486

**Published:** 2023-05-15

**Authors:** Sopee POOMSAWAT, Abdulshukor KARIYA, Thirayost NIMMANON, Theerachai KOSANWAT, Rachai JUENGSOMJIT, SANGUANSIN Sirima

**Affiliations:** 1 Mahidol University Faculty of Dentistry Department of Oral and Maxillofacial Pathology Bangkok Thailand Mahidol University, Faculty of Dentistry, Department of Oral and Maxillofacial Pathology, Bangkok, Thailand.; 2 Yala Regional Hospital Anatomical Pathology Unit Yala Thailand Yala Regional Hospital, Anatomical Pathology Unit, Yala, Thailand.; 3 Phramongkutklao College of Medicine Department of Pathology Bangkok Thailand Phramongkutklao College of Medicine, Department of Pathology, Bangkok, Thailand.; 4 Mahidol University Faculty of Dentistry Department of Oral Biology Bangkok Thailand Mahidol University, Faculty of Dentistry, Department of Oral Biology, Bangkok, Thailand.

**Keywords:** Dysplasia, Oral leukoplakia, Oral potentially malignant disorders, Oral squamous cell carcinoma, Type VII collagen

## Abstract

Type VII collagen (Col7) is a major component of anchoring fibrils. Col7 plays a role in tumor development and aggressiveness of cutaneous squamous cell carcinoma of recessive dystrophic epidermolysis bullosa. However, the role of Col7 in oral squamous cell carcinoma (OSCC) and oral leukoplakia (OL) remains largely unknown.

**Objective:**

To elucidate the role of Col7 and its diagnostic potential during oral carcinogenesis.

**Methodology:**

Col7 expression was immunohistochemically studied in 254 samples, including normal oral mucosa (NM), OL without dysplasia, OL with dysplasia, and OSCC. The correlation between Col7 expression and clinicopathologic parameters of OSCC was also determined.

**Results:**

Col7 was present as a linear deposit at the basement membrane of NM, OL without dysplasia and OL with dysplasia, and at the tumor-stromal junction around tumor islands in OSCC. Discontinuity of expression was frequently observed in OL with dysplasia and OSCC. OSCC had the significantly lowest Col7 expression (p<0.0001). Compared with OL without dysplasia, OL with dysplasia showed significantly reduced Col7 expression. Patients in clinical stage 4 with positive nodes had low Col7 expression compared with those in clinical stage 1 and negative nodes, respectively.

**Conclusion:**

Loss of Col7 is associated with tumorigenesis and aggressiveness in OSCC. A significantly reduced Col7 expression in OSCC implies that Col7 may be a useful marker for diagnosis and therapeutic targets.

## Introduction

Type VII collagen (Col7) is the major component of anchoring fibrils, a device securing the basement membrane to the underlying connective tissue.^1^ In the lamina densa, Col7 binds to basement membrane proteins, including laminin-332 and type IV collagen, while interacting with collagen fibrils in the sublamina densa.^2-4^ Col7 is found in the basement membrane zone of several organs such as the skin, oral mucosa, esophagus, larynx, breast, prostate, and colon.^5,6^ The crucial function of Col7 in the integrity of the epithelial-connective tissue interface is well demonstrated in recessive dystrophic epidermolysis bullosa (RDEB). Patients with RDEB have mutations in the *COL7A1* gene encoding for Col7, leading to an absence or drastic reduction of Col7 and numbers of anchoring fibrils.^7^ These patients have chronic skin and mucosal fragility, blistering, and experience repeated cycles of wound and repair.^8^ Importantly, nearly all patients with severe RDEB develop cutaneous squamous cell carcinoma (SCC) during their teenage years and 80% die from metastatic SCC.^9^ Additionally, an increased risk for cutaneous SCC is also found in other subtype of RDEB.^9,10^ The observations described in cutaneous SCC suggest that depletion of Col7 promotes tumorigenesis and aggressive behavior in this cancer. However, the non-collagenous domain (NC1) of Col7 is required for tumor formation by RDEB keratinocytes,^11^ showing that Col7 plays an important role in cutaneous SCC, but the loss or gain of Col7 needed for tumor development and aggressiveness remains controversial.

Col7 may function in a cancer-specific manner. While Col7 was upregulated in cancers from the colon and esophagus,^12,13^ it was downregulated in cancers derived from the lungs, breast, and larynx.^14-16^ Recently, in gastric cancer, studies showed that patients with intracellular Col7 expression had lower five-year overall survival than those with only extracellular expression.^17^ The effect of Col7 on patient outcomes in esophageal and laryngeal SCC also showed conflicting results. In esophageal SCC, the five-year survival rate of patients with negative Col7 or low level of mRNA was better than that of patients with positive tumors or high level of mRNA.^12,18^ By contrast, laryngeal SCC and dysplastic lesions showed loss or disruptions of Col7 expression. Furthermore, Col7 expression was inversely correlated to tumor size, lymph node metastasis, and local recurrence. Because laryngeal SCC showed statistically significant defects in Col7 staining compared to dysplastic lesions, Col7 was proposed to be a marker for early invasion in laryngeal SCC.^19^ Although the clinical significance of Col7 expression in SCC from different organs appears to be in conflict, Col7 expression in SCC samples of various organs is rarely investigated. To date, only two studies have analyzed the Col7 expression in oral SCC (OSCC). In a series of benign and malignant tumors, Wetzels, et al.^5^ (1992) showed that Col7 was present at basement membranes surrounding the tumor islands of all 23 cases of OSCC. Using *in situ* hybridization and immunohistochemistry, Kainulainen, et al.^20^ (1997) investigated Col7 expression in 22 cases of OSCC and concluded that Col7 was highly synthesized in OSCC, but its distribution at the basement membranes surrounding tumor islands was impaired; seven cases of oral epithelial dysplasia were also studied by in situ hybridization in Kainulainen’s study.^20^ The signal for Col7 mRNA in oral epithelial dysplasia was uneven compared to normal oral mucosa. The expression of Col7 protein and mRNA in dysplasia and SCC from larynx and oral mucosa suggests that Col7 plays a significant role during both the early and late stages of carcinogenesis. However, the role of Col7 in OSCC remains largely unknown. Furthermore, Col7 expression has never been studied in oral leukoplakia (OL). OL is a predominantly white lesion of the oral mucosa and is one of the most important oral potentially malignant disorders. It is well established that many OSCC cases arise from OL.^21^ A recent meta-analysis showed that the malignant transformation rate of OL is 9.7%.^22^ Thus, OL is considered the early state of oral carcinogenesis.

Our study aimed to elucidate the role of Col7 and its diagnostic potential during the initial and final events in oral carcinogenesis. We investigated Col7 expression by immunohistochemistry in OL without dysplasia, OL with dysplasia, and OSCC using normal oral mucosa as controls. Col7 expression between these four tissue types was subsequently compared. Additionally, to assess the clinical significance of Col7 in OSCC, we examined the correlation between Col7 expression and clinicopathologic parameters of OSCC.

## Methodology

### Tissue samples

This study was approved by the Institutional Review Boards (MU-DT/PY-IRB 2018/051.0211 and IRBTA0057/2022) in compliance with the ethical standards of the 1964 Helsinki Declaration and its later amendments or comparable ethical standards. Cases of clinically diagnosed OL were retrieved from departmental archives. Then, hematoxylin and eosin stained sections of these cases were reevaluated by a board certified oral pathologist (SP). OL samples were grouped as OL without dysplasia and OL with dysplasia depending on the presence of epithelial dysplasia. The architectural and cytological disturbances were used to decide the presence of epithelial dysplasia. The severity of epithelial dysplasia was classified into mild, moderate, and severe groups.^23^ Cases with histopathologic diagnosis of OSCC were retrieved. Among OSCC subjects, clinicopathologic data, including age, sex, location, TNM category, clinical staging, histopathologic grade, tumor depth of invasion, perineural invasion, and lymphovascular invasion were recorded. The TNM system and clinical staging in this study were based on the latest WHO classification of carcinomas of the lip and oral cavity.^23^

Selected cases were retrieved as formalin-fixed, paraffin-embedded blocks. Samples of normal oral mucosa were obtained from patients or healthy volunteers undergoing minor surgery. All specimens of normal oral mucosa were fixed in 10% buffered formalin, processed, and embedded in paraffin blocks under the same conditions as paraffin blocks of OL and OSCC.

### Immunohistochemistry

Immunohistochemistry was performed using formalin-fixed, paraffin-embedded sections, as previously described, with minor modifications.^24^ 4-µm-thick sections were cut and placed on glass slides coated by aminopropyltriethoxysilane (Sigma Chemical Co., St Louis, MO, USA). Sections were dewaxed and rehydrated. Endogenous peroxidase activity was suppressed by incubating the sections in 3% H_2_O_2_ for 10 minutes. Antigen retrieval was achieved by heating the slides in 10mM citrate buffer pH 6.0 and trypsin solution (pH 7.8). The sections were cooled for 1 hour. After washing with 0.1% Tween 20 (MERCK-Schuchardt, Hohanbrunn, Germany) in phosphate buffered saline (PBS), the sections were treated with 5% bovine serum albumin (Sigma Chemical Co.) in PBS for 30 minutes. Then, the sections were covered with mouse monoclonal antibody raised against Col7 (clone LH7.2; diluted 1:50; Santa Cruz Biotechnology, Inc, CA, USA) for 2 hours. The monoclonal antibody clone LH7.2 is directed against the non-helical carboxy terminal region of Col7.^25^ After rigorous washing in 0.1% Tween 20 in PBS, labeled polymer (Dako Envision System, Dako Corporation, Carpinteria, CA, USA) was applied to the sections for 30 minutes. After final washing with PBS, freshly made diaminobenzidine (Sigma Chemical Co) was used to visualize the immunoreactivity. Sections were then briefly washed in running tap water and counterstained with Mayer’s hematoxylin.

For each specimen, negative control was performed by omitting the primary antibody. After trial and error, sections of a pyogenic granuloma, demonstrating a continuous, linear golden brown staining at the basement membrane zone were chosen to serve as positive controls for each run. All sections were processed under identical protocols.

### Assessment of immunohistochemical Col7 expression

Col7 expression was semi-quantitatively evaluated under a microscope. First, the amount of immunoreactivity along the basement membrane zone of the normal oral mucosa, OL without dysplasia, OL with dysplasia, and around tumor islands of OSCC was classified according to a score of 0 (expression less than 5%), 1 (expression between 5 and 25%), 2 (expression between 26 to 50%), and 3 (expression more than 51%). Second, the staining intensity was scored as 0 (no staining), 1 (weak intensity), 2 (moderate intensity), and 3 (strong intensity). After calibration, all sections were independently investigated by SP (a board certified oral pathologist) and AK (a board certified pathologist). Sections in disagreement of evaluation were discussed and reevaluated until consensus was reached. Finally, the staining index of each case was calculated by multiplying the grade of expression by the score of staining intensity. The staining index of each case was given a score ranging from “0” to “9”.

### Statistical analysis

The comparisons of the staining index of Col7 between the four tissue types were conducted using the Kruskal-Wallis test followed by Dunn’s multiple comparison test.

To evaluate the relationship between Col7 expression and clinicopathologic parameters, the Mann-Whitney test was used. Statistically significant differences were designated at p<0.05. All statistical analyses were performed in GraphPad Prizm 8.2.0 for Windows, GraphPad Software, San Diego, CA, USA.

## Results

### Characteristics of samples

Col7 expression was studied among 254 samples. They comprised 16 cases of normal oral mucosa, 46 cases of OL without dysplasia, 103 cases of OL with dysplasia, and 89 cases of OSCC. In the normal oral mucosa group, 9 subjects were women and 6 subjects were men. The average age in this group was 43.3 years, ranging from 15 to 83 years. The age and sex of one subject in the normal oral mucosa group were unavailable. Tables 1 and 2 shows the demographic data of OL without dysplasia, OL with dysplasia, and OSCC, respectively.

**Table 1:** Demographic data of OL without dysplasia and OL with dysplasia

Characteristic	Category	OL without dysplasia	OL with dysplasia
		**Cases (%)**	**Cases (%)**
Sex	Male	22 (47.8)	38 (36.8)
	Female	24 (52.2)	65 (63.1)
Age	Average (range)	57.2 (28-83)	61.7^#^ (28-90)
Degree of epithelial dysplasia	Mild	-	38 (36.9)
	Moderate	-	42 (40.8)
	Severe	-	23 (22.3)
Location	Buccal mucosa	10 (21.74)	31 (30.10)
	Palate	5 (10.87)	24 (23.30)
	Tongue	6 (13.04)	22 (21.36)
	Gingiva	11 (23.91)	12 (11.65)
	Alveolar mucosa	8 (17.39)	6 (5.83)
	Lower lip	-	4 (3.88)
	Retromolar	6 (13.04)	2 (1.94)
	Upper lip	-	1 (0.97)
	Floor of mouth	-	1 (0.97)

^#^The ages of the five cases are not available

**Table 2:** Demographic data of OSCC

Characteristic	Category	Cases (%)
Sex	Male	54 (60.7)
	Female	35 (39.3)
Age	Average (range)	56 (27-85)
Histopathologic differentiation	Well	43 (48.3)
	Moderate	44 (49.4)
	Poor	2 (2.2)
Location	Tongue	54 (60.67)
	Floor of the mouth	12 (13.48)
	Palate	6 (6.74)
	Buccal mucosa	5 (5.62)
	Gingiva	4 (4.49)
	Retromolar	4 (4.49)
	Lower lip	3 (3.37)
	Upper lip	1 (1.12)

### Pattern of Col7 staining in the four tissue types

**Figure 1:**
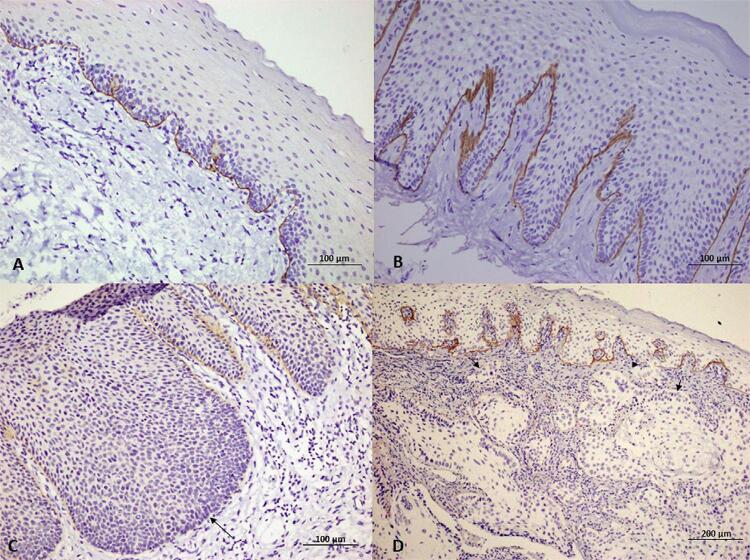
Expression of type VII collagen (Col7) in representatives of normal oral mucosa (a), oral leukoplakia (OL) without dysplasia (b), OL with dysplasia (c), and oral squamous cell carcinoma (d). In normal oral mucosa (a) and OL without dysplasia (b), Col7 is expressed as a continuous linear deposit at the basement membrane zone. In OL with dysplasia (c), Col7 is also expressed as a linear deposit along the basement membrane zone, but loss of expression is also observed (arrow). In oral squamous cell carcinoma (d), weak staining can be detected at the junction between tumor islands and stroma. Loss of expression (arrows) is also observed in some areas. Notably, the histologically normal covering epithelium shows a strong linear deposit at the basement membrane zone. Absence of expression is noticed mainly at the invasive area (arrowheads)

In all samples of normal oral mucosa, Col7 was present as a continuous linear deposit at the junction between the epithelium and the underlying connective tissue or at the basement membrane zone (Figure 1a). The staining intensity varied from moderate to strong. Col7 expression in the OL without dysplasia group was usually similar to those of the normal oral mucosa. Approximately 98% of OL without dysplasia showed continuous linear deposits with moderate to strong staining intensity at the basement membrane zone (Figure 1b). A focal loss of staining was noticed in only one case. Unlike the normal oral mucosa and OL without dysplasia groups, continuity of Col7 at the basement membrane zone was less frequently observed in the OL with dysplasia group; in this group, an intact Col7 at the basement membrane zone was noted in 67%, whereas small to large interruptions were discovered in 33% (Figure 1c). Additionally, 31% of OL with dysplasia cases showed weak staining intensity, whereas weak staining was not found in any samples of the normal oral mucosa and OL without dysplasia groups.

**Figure 2:**
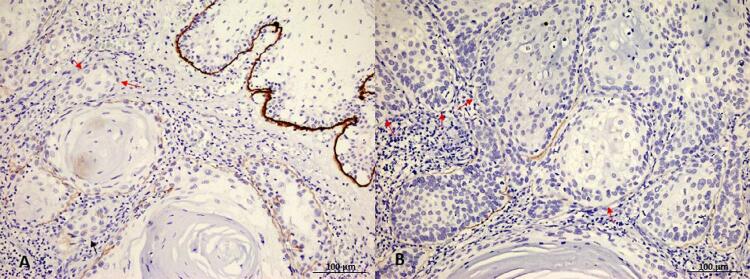
Expression of type VII collagen (Col7) in representatives of oral squamous cell carcinoma (OSCC) (a), Col7 is weakly expressed around tumor islands whereas it is strongly expressed at the basement membrane zone of the covering epithelium. Note that loss of Col7 is observed in some areas (red arrows) (b), In this particular OSCC, loss of Col7 is frequently observed (red arrows), although weak to moderate staining can be detected around a few tumor islands

In the OSCC group, complete loss of Col7 expression was found as high as 70.8% (63/89 cases). For all 26 positive cases, weak to strong staining intensity was identified as linear deposits around tumor islands (Figure 1d and Figure 2). No other staining patterns such as nuclear or cytoplasmic staining were observed. A total of nine cases (10.1%) displayed continuous linear deposits whereas the remainder 17 cases (19.1%) exhibited moderate to substantial loss of expression; additionally, irregular or fragmentary staining was noted. In some specimens, a histologically normal covering epithelium was also present along with the invading tumors. This covering epithelium, serving as a good internal positive control, showed a moderate linear deposit of Col7 at the basement membrane zone (Figures 1d and 2a).

### Staining index of Col7 in the four tissue types.

Col7 expression between the four tissue types was compared using the staining index. In each specimen, this index was estimated by multiplying the degree of Col7 expression by the staining intensity. The staining index of Col7 in the four tissue types is summarized in Table 3. A significantly reduced staining index of Col7 was detected in OSCC compared to the normal oral mucosa, OL without dysplasia, and OL with dysplasia (p<0.0001). Additionally, OL with dysplasia showed significantly reduced Col7 compared with OL without dysplasia (p=0.0005). In the OL with dysplasia group, a comparison of staining index between different classifications of epithelial dysplasia showed no significant differences (p=0.1532).

**Table 3:** Staining index of Col7 among the four tissue types

Tissue type	Number of cases	Median (Interquartile range)
Normal oral mucosa	16	6 (6-9)*
Oral leukoplakia without dysplasia	46	9 (6-9)*^#^
Oral leukoplakia with dysplasia	103	6 (3-9)*
Oral squamous cell carcinoma	89	0 (0-1.5)

*p<0.0001, statistically significant difference compared with oral squamous cell carcinoma.

#p=0.0005, statistically significant difference compared with oral leukoplakia with dysplasia.

### Correlation between Col7 expression and clinicopathologic parameters in OSCC

In this study, we also examined the correlation between Col7 expression and clinicopathologic parameters including age, sex, location, clinical stages, tumor size, lymph node involvement, histopathologic grade, depth of invasion, and perineural and lymphovascular invasion. Table 4 shows Col7 expression regarding these clinicopathologic features. The Col7 expression significantly correlated to clinical stage (p=0.0258) and regional lymph node involvement (p=0.0174). Patients with clinical stage 4 had low Col7 expression compared to patients at stage 1. Additionally, patients with positive nodes had low Col7 expression compared to those of negative nodes. No significant correlations were observed between Col7 expression and other clinicopathologic factors.

**Table 4:** Correlation between staining index of Col7 and the clinicopathologic parameters of 89 patients with OSCC

Clinicopathologic parameters	No. of patients (%)	Median (IQR)	p value#
**Age (years)**			
<60	60 (67.4)	0 (0-0)	0,1531
≥60	29 (32.6)	0 (0-2)	
**Sex**			
Male	54 (60.7)	0 (0-1)	0,654
Female	35 (39.3)	0 (0-2)	
**Localization**			
Tongue	54 (60.7)	0 (0-1.25)	0,8367
Non-tongue	35 (39.3)	0 (0-2.0)	
**Clinical stage**^**a**^			
Stage 1	12 (14.0)	2.5 (0-5.5)	0.0258*
Stage 2	19 (22.1)	0 (0-2)	
Stage 3	20 (23.3)	0 (0-0.75)	
Stage 4	35 (40.7)	0 (0-0)	
**T classification**^**b**^			
T1	19 (21.8)	0 (0-3)	0,7189
T2	27 (31.0)	0 (0-1)	
T3	17 (19.5)	0 (0-1)	
T4	24 (27.6)	0 (0-1.5)	
**Regional lymph node involvement**^**a**^			
Negative	42 (48.8)	0 (0-3)	0.0174*
Positive	44 (51.2)	0 (0-0)	
**Histopathologic grade**			
Well	43 (48.3)	0 (0-1)	0,4639
Moderate + Poor	46 (51.7)	0 (0-2)	
**Depth of invasion**^**c**^			
≤5 mm	42 (47.2)	0 (0-1.25)	0,8214
>5 mm and ≤10	26 (29.2)	0 (0-0.25)	
>10 mm	20 (22.5)	0 (0-2)	
**Perineural invasion**^**b**^			
Not present	63 (70.8)	0 (0-2)	0,2107
Present	24 (30.0)	0 (0-0)	
**Lymphovascular invasion**^**b**^			
Not present	76 (85.4)	0 (0-1.75)	0,3278
Present	11 (12.4)	0 (0-0)	

*statistically significant

^#^Kruskall-Wallis H test or Mann-Whitney U test

^a^86 cases. ^b^87 cases. ^c^88 cases

IQR=interquartile range

## Discussion

This study showed the significantly reduced Col7 in OSCC compared with normal oral mucosa, OL without dysplasia, and OL with dysplasia. Additionally, OL with dysplasia showed significantly reduced Col7 compared to OL without dysplasia. The correlation between Col7 expression and clinicopathologic factors of OSCC showed that patients with clinical stage 4 and positive nodes had low Col7 expression compared to those of clinical stage 1 and negative nodes.

Few studies investigate Col7 expression in normal oral mucosa.^5,26-28^ Eversole, et al.^29^ (1994) used buccal mucosa obtained from histologically normal tissue from fibromas, mucoceles, and keratoses, while Raminez-Amador, et al.^30^ (1996) used normal mucosa from histologically normal tissue of unaffected buccal mucosa in oral patients with lichen planus. Haapalainen, et al.^31^ (1995) do not mention the method to obtain normal oral mucosa in their study. Therefore, we compared our results only with genuine normal oral mucosa. Consistent with former studies,^5,26-28^ we found that Col7 was exclusively located at the basement membrane zone as continuous thin to thick linear deposits. Col7 was also expressed at the basement membrane of stratified squamous epithelium of other tissues including the esophagus, larynx, trachea, ectocervix, and skin.^5,6^ Previous studies showed that unlike type IV collagen, Col7 was not present around blood vessels.^6,15,31^ The staining pattern of Col7 in normal oral mucosa found in this study indicates that the immunohistochemical technique in our study was properly performed.

Col7 expression in OL, the most common oral potentially malignant disorder, has never been reported. Similar to normal oral mucosa, OL without dysplasia showed a continuous linear deposit of Col 7 at the basement membrane zone. However, in OL with dysplasia, only 67% showed an intact Col7 expression, whereas 33% displayed small to large defects. Similar to our study, only 50% of dysplastic larynx cases showed intact Col7 expression.^19^ It is noteworthy that the staining index of Col7 significantly differed between OL with and without dysplasia. These results suggest that Col7 is involved in the early event of oral carcinogenesis and may be a useful marker to assist in diagnosing oral epithelial dysplasia. Loss of Col7 may be a consequence of failure to properly synthesize Col7 by dysplastic keratinocytes or a result from Col7 high degradation rate. A previous study showed that the mRNA of Col7 in epithelial dysplasia is uneven compared to normal oral mucosa, as some basal cells showed strong signals, whereas other cells exhibited weak expression.^20^ Thus, the loss of Col7 in OL with dysplasia is likely the consequence of the inability to maintain proper production of Col7 by dysplastic keratinocytes. Additionally, this characteristic may be a key feature of premalignant keratinocytes.

Our result regarding the disruption of Col7 in OL with dysplasia agrees with the findings in dysplastic larynx.^19^ All basement membrane components showed defects when using immunohistochemistry for investigation: Col7, type I collagen, laminin-111, heparansulfate proteoglycan, and fibronectin; however, it was Col7 that showed significant defects, highlighting its crucial role in dysplastic process. Interestingly, two cases of dysplastic larynx developed invasive carcinoma within six to 12 months. These results imply that Col7 has a high potential to be a biomarker for early detection of SCC. Perhaps this prognostic role of Col7 in larynx exists in the oral mucosa. Therefore, it is of interest to study the malignant transformation of OL with dysplasia that had low staining index of Col7.

Based on the English literature on the subject, only two studies conducted in 1992 and 1998 investigated Col7 expression in OSCC.^5,20^ Therefore, the role of Col7 in OSCC remains largely unknown. This study found that Col7 expression was exclusively located at the tumor-stromal interface with various degrees of disruptions, and this staining pattern may have somewhat differed from two previous groups.^5,20^ The finding that Col7 was located at the tumor-stromal interface is consistent with two earlier studies. However, a cytoplasmic staining of some peripheral tumor cells was also discovered in 18 of 22 cases in Kainulainen, et al.^20^ (1997) and in 6 of 23 cases in Wetzel, et al.^5^ (1992). We found that the cytoplasmic staining in a photo provided in Kainulainen’s work^20^ was difficult to observe. Additionally, a slight granular staining of blood vessels was also detected in the study by Wetzel, et al. (1992).^5^ The staining around blood vessels may be unusual for Col7 as other studies showed that Col7 was not expressed at this location.^6,15,31^ Col7 expression in SCC from the larynx and esophagus also showed a different staining pattern. In SCC from the larynx, Col7 was localized at and restricted to the tumor-stromal interface. However, SCC from the esophagus showed predominant cytoplasmic staining. The basement membrane-like pattern and the nuclear reactivity of malignant cells at the invasive front were also detected in esophageal SCC. We speculate that the nuclear staining and the staining around blood vessels in these related studies may be a cross reactivity to other antigens. Thus, cytoplasmic staining in the peripheral cells may also relate to the cross reactivity. These contradictory results may be partially explained by the differences in immunohistochemical techniques and sources of primary antibodies. However, it would still be possible that Col7 of SCC from different organs may be expressed differently.

The comparison of Col7 expression between normal oral mucosa, OL without dysplasia, OL with dysplasia, and OSCC has never been investigated. Our study found that Col7 expression in OSCC was significantly lower than those of normal oral mucosa and OL with and without dysplasia. These results clearly suggest the key role of Col7 in OSCC. Loss of Col7 is also an important characteristic of OSCC and Col7 may be a good marker for diagnosing OSCC. Additionally, Col7 may be a suitable therapeutic target for OSCC.^32^ The finding that Col7 expression of OSCC was significantly lower than OL with dysplasia implies that loss of Col7 is an important step in transforming the noninvasive to invasive status in OSCC. These results are consistent with the observations conducted in human laryngeal samples.^19^ As Col7 provides the stability for the natural barrier basement membrane, loss of Col7 at this location may allow dysplastic keratinocytes to invade the underlying connective tissue. Moreover, loss of Col7 may actively promote tumorigenesis in OSCC. This concept is supported by the results in DMBA-TPA carcinogenesis experiments; hypomorphic mice with only 10% Col7 expression developed more invasive tumors compared to the wild-type mice.^33^ Interestingly, lack of Col7 in keratinocytes increases the levels of cathepsin B and Z, important lysosomal proteases that promote tumorigenesis in various mouse models of human cancers.^34,35^ Additionally, decreased Col7 expression in RDEB keratinocytes promotes keratinocyte motility and increases expression of matrix metalloproteinase (MMP)-1, MMP-2, MMP-3, and MMP-9;^36^ all events contribute to cell invasion. However, loss of Col7 in OSCC may be a bystander effect. Thus, further investigations are needed to clarify if loss of Col7 is actively involved in tumor progression in OSCC.

A correlation between Col7 expression in OSCC and their clinicopathologic parameters has never been demonstrated. Interestingly, loss of Col7 expression was significantly related to clinical status and lymph node involvement. Patients with advanced clinical stage and positive lymph nodes had extensive loss of Col7 expression compared to those of clinical stage 1 and negative nodes. Our results agree with a related investigation in laryngeal SCC.^15^ Patients with large tumor sizes and positive nodes had more defects of Col7 than patients with small tumors and negative nodes. These results suggest that Col7 is implicated in the aggressive behavior of SCC from oral mucosa and larynx. Supporting this notion, analysis from the whole-genome Illumina expression arrays and immunostaining discovered that loss of Col7 elevates the expression of the chemokine ligand-receptor CXCL10-CXCR3 and downstream-associated phospholipase C signaling, which may increase metastatic potential in SCC.^37^ CXCL10 and its receptor CXCR3 promoted lymph node metastasis in melanoma and colon cancer^38,39^ and lung metastasis in breast cancer.^40^ Additionally, Col7 knockdown SCC keratinocytes exhibited several characteristics of aggressive behaviors such as increased cell migration and invasion, increased MMP-2 activities, induced epithelial-mesenchymal transition, decreased terminal differentiation, and enhanced transforming growth factor-β signaling.^37,41^ Angiogenesis induced by loss of Col7 was observed in SCC in mouse xenografts and RDEB tumors. Interestingly, in a mouse model, this enhanced angiogenesis was reversed by human recombinant Col7 protein therapy.^41^

## Conclusion

In conclusion, Col7 expression was interrupted in OL with dysplasia and significantly reduced in OSCC. A significant loss of Col7 expression was observed among patients with advanced clinical stage and positive nodes. These results suggest that Col7 not only plays a crucial role during the early and late stages of oral carcinogenesis but is also implicated in the aggressive behavior of OSCC. In OSCC, we believe that the loss, not gain, of Col7 is associated with tumorigenesis and aggressiveness. A significantly reduced Col7 expression in OSCC compared with normal oral mucosa, OL without dysplasia, and OL with dysplasia implies that Col7 may be a useful marker for diagnosis and therapeutic targets.
